# Neoadjuvant afatinib in a patient with locally advanced lung adenocarcinoma harboring an NPTN-NRG1 fusion: a case report

**DOI:** 10.1186/s12957-026-04233-6

**Published:** 2026-03-14

**Authors:** Chu-Yu Zhou, Zi-Yi Xu, Zhen-Bin Qiu, Fei Wang, Hong-Ji Li, Ri-Qiang Liao, Yi-Long Wu, Wen-Zhao Zhong

**Affiliations:** Guangdong Lung Cancer Institute, Guangdong Provincial Key Laboratory of Translational Medicine in Lung Cancer, Guangdong Provincial People’s Hospital, Guangdong Academy of Medical Sciences, Southern Medical University, Guangzhou, China

**Keywords:** Non-small cell lung cancer, Neoadjuvant therapy, Afatinib, Single-cell RNA sequencing, T cell

## Abstract

**Background:**

Stage III non-small cell lung cancer (NSCLC) is heterogeneous and poses significant treatment challenges. In recent years, neoadjuvant therapy has emerged as a promising treatment strategy for patients with stage III NSCLC.

**Case presentation:**

We report the case of a 39-year-old woman with stage IIIB lung adenocarcinoma harboring a neuroplastin-neuregulin 1 (NPTN**-**NRG1) fusion who achieved a dramatic response to neoadjuvant afatinib. After three months of therapy, imaging showed a partial response; the patient subsequently underwent complete surgical resection with an uneventful recovery and has remained disease-free to date. To explore the mechanism underlying this response, we performed single-cell RNA sequencing (scRNA-seq) and T-cell receptor (TCR) sequencing of tumor tissues and lymph nodes. The analysis revealed a mutual interaction between CD8^+^ effector T cells, CD8^+^ memory T cells, and natural killer-like (NK-like) T cells via C-C chemokine ligand (CCL)5-C-C chemokine receptor (CCR)5, CCL5-CCR3, and CCL5-CCR1 ligand-receptor pairs. These findings indicate that the post-treatment microenvironment is characterized by active chemokine signaling and immune cell crosstalk, which may contribute to the anti-tumor response.

**Conclusions:**

This case highlights the efficacy of afatinib in the perioperative treatment of NSCLC with NRG1 fusion, and supports its potential as a personalized therapeutic strategy.

**Supplementary Information:**

The online version contains supplementary material available at 10.1186/s12957-026-04233-6.

## Background

Neuregulin 1 (NRG1) gene fusions are rare oncogenic drivers of NSCLC, occurring in approximately 0.3% of cases [1–5]. These fusions, most often reported in invasive mucinous adenocarcinomas, ectopically express an epidermal growth factor-like domain that activates erb-b2 receptor tyrosine kinase (ERBB) 3, which in turn heterodimerizes with ERBB2 to drive oncogenic Phosphatidylinositol 3-kinase/ protein kinase B and mitogen-activated protein kinase signaling [1, 6]. Because NRG1 fusions are uncommon and involve diverse partner genes, their biological behaviors and optimal therapies remain poorly defined. Pan-ErbB-family tyrosine kinase inhibitors (TKIs) such as afatinib have shown clinical activity in selected NRG1 fusion-positive cases [7–9]. However, to the best of our knowledge, afatinib has not been previously reported as a neoadjuvant therapy for locally advanced NSCLC with the NPTN**-**NRG1 fusion. Herein, we describe this case, emphasizing its unique features, clinical relevance, and associated tumor immune response.

## Case presentation

**Fig. 1:**
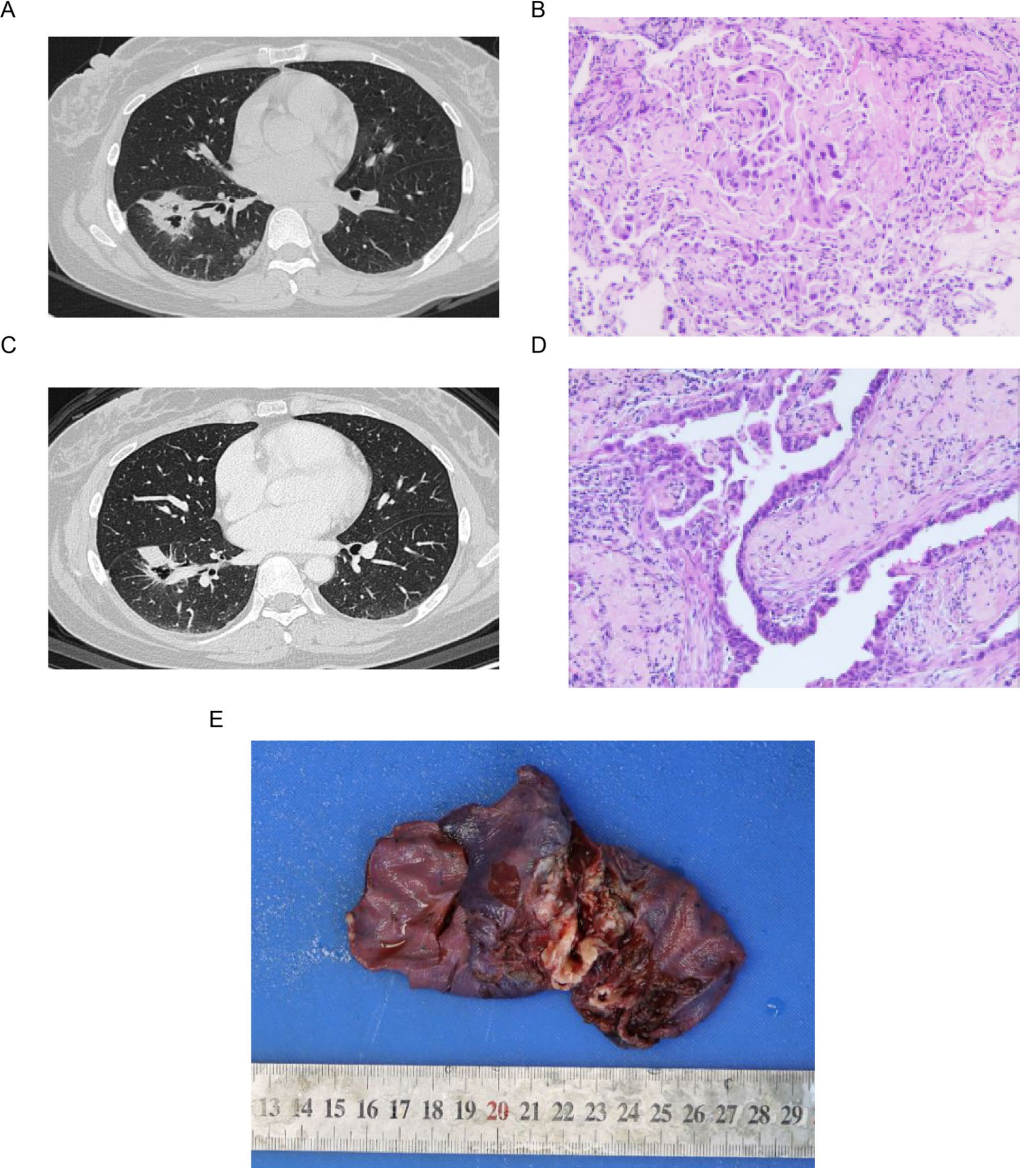
Neoadjuvant afatinib response. (**A**) Contrast-enhanced chest CT scans at baseline. (**B**) H&E-stained bronchoscopic biopsy specimen obtained before therapy, confirming lung adenocarcinoma. (**C**) Contrast-enhanced chest CT scans after three months of afatinib therapy, showing marked tumor regression. (**D**, **E**) Resected tumor and the gross specimen of the right lower lung lobe following three months of treatment. CT, computed tomography

A 39-year-old woman with no history of smoking presented with a two-week history of productive cough. Contrast-enhanced chest computed tomography (CT) revealed a 3.2 cm mass in the right lower lobe and enlargement of the right supraclavicular, right hilar, and mediastinal lymph nodes (Fig. 1A). Brain magnetic resonance imaging and bone scans showed no evidence of distant metastasis. Bronchoscopic biopsy confirmed an invasive lung adenocarcinoma (Fig. 1B), which was clinically staged as T2aN3M0 (stage IIIB) according to the 8th edition of the Union for International Cancer Control TNM staging system. Because the bronchoscopic specimen was insufficient for genetic analysis, the right supraclavicular lymph node was excised for molecular testing. DNA- and RNA-based next-generation sequencing (NGS) of 654 cancer-related genes identified an NPTN-NRG1 fusion with no other activating driver mutations. After a multidisciplinary discussion, neoadjuvant therapy was recommended to achieve tumor downstaging before surgery.

Neoadjuvant afatinib (40 mg daily) was initiated after obtaining informed consent. After three months of therapy, restaging contrast-enhanced CT demonstrated a partial response according to the Response Evaluation Criteria in Solid Tumors 1.1 criteria (Fig. 1C). The patient experienced treatment-related adverse events classified according to the Common Terminology Criteria for Adverse Events version 5.0, including Grade 3 skin rash, Grade 1 fatigue, and Grade 2 oral ulceration. All adverse events were managed supportively.

After three months of treatment, the patient underwent a right lower lobectomy with systematic lymphadenectomy (Fig. 1D-E). Surgery and recovery were uneventful. Postoperative pathological assessment was independently reviewed by two experienced pathologists in strict accordance with the IASLC Multidisciplinary Recommendations for Pathologic Assessment of Lung Cancer Resection Specimens After Neoadjuvant Therapy. Quantitative evaluation of the primary tumor bed revealed approximately 35% residual viable tumor, 5% necrosis, and 60% tumor stroma (comprising fibrosis, histiocytes and lymphocytes). Consequently, the patient did not achieve a Major Pathologic Response. Additionally, viable tumor cells were identified in lymph node stations 2, 7, and 12. Peripheral blood circulating tumor DNA (ctDNA) testing was negative before and after surgery. Molecular analysis of the surgical specimen confirmed the preoperative findings, and the DNA/RNA NGS panel still detected the NPTN-NRG1 fusion. However, a broader DNA panel (1021 genes) on the resected tissue showed only a variant in Fc gamma receptor IIb (*FCGR2B*), with no other known driver alterations.

Because of earlier treatment-related toxicity, adjuvant afatinib was continued at a reduced dose of 30 mg daily. The planned duration of adjuvant therapy is 3 years. At 7 months post-surgery, contrast-enhanced chest CT showed no evidence of residual or recurrent disease.

**Fig. 2:**
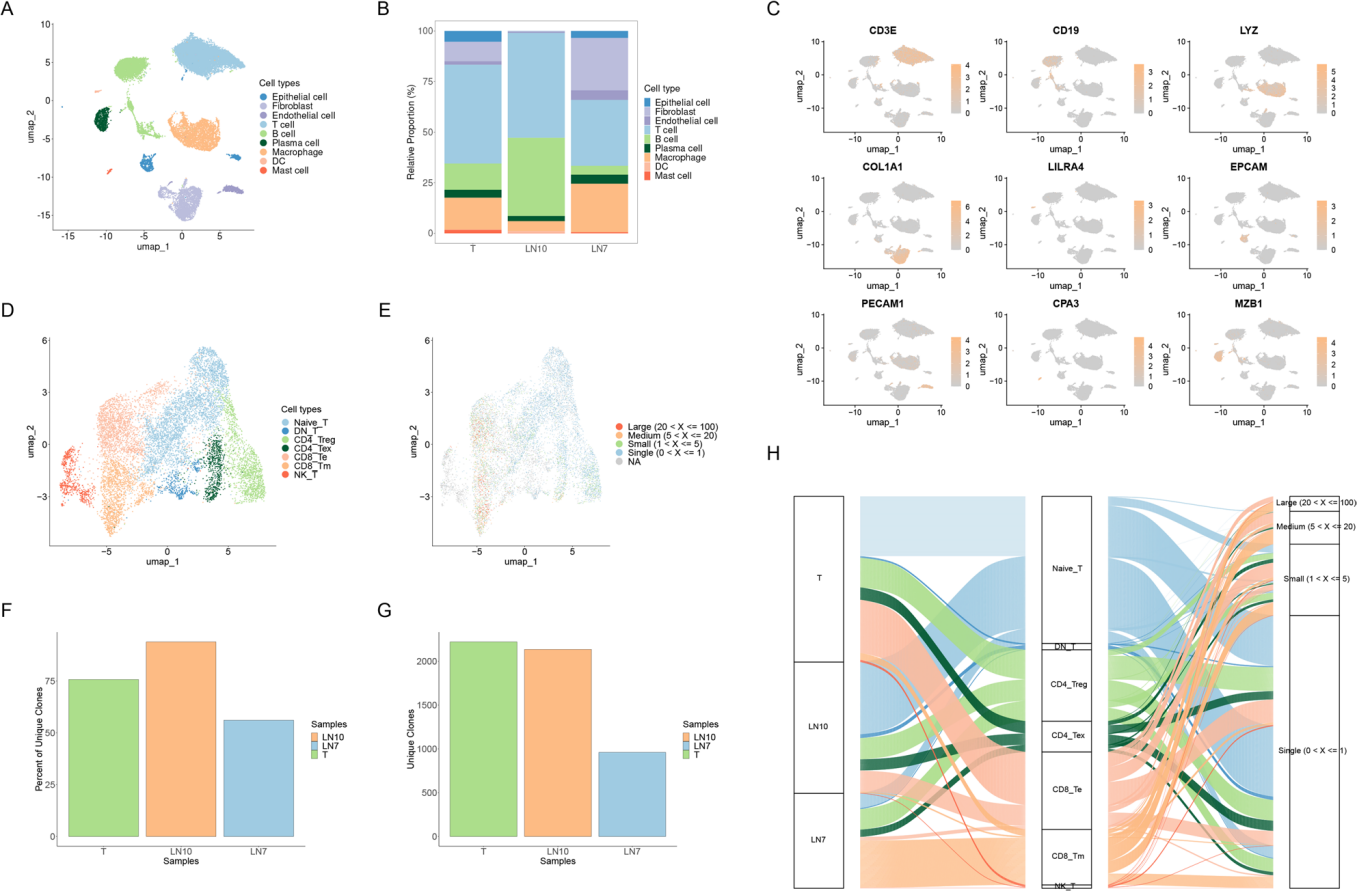
Single-cell RNA-seq and TCR-seq of tumor and lymph nodes revealing the post-treatment cellular and immunological landscape. (**A**) UMAP plot of all cells showing nine distinct cell types. (**B**) Bar chart of cell type composition in the tumor (T), hilar lymph nodes (LN10), and subcarinal lymph nodes (LN7). (**C**) Feature plots of canonical marker gene expression across the identified cell types. (**D**) UMAP plot highlighting seven T cell subtypes. (**E**) UMAP plot illustrating TCR clonal expansion. (**F**, **G**) Bar plots of the unique TCR clone% (**F**) and count (**G**) in each sample. (**H**) Alluvial diagram illustrating the distribution of TCR clones across sample origin, T cell subtype, and clonal expansion level. TCR, T-cell receptor; UMAP, uniform manifold approximation and projection

To explore treatment-induced changes in the tumor microenvironment, we performed single-cell RNA sequencing (scRNA-seq) and T-cell receptor (TCR) sequencing of the resected tumor and lymph node samples. Detailed methods regarding single-cell sequencing and bioinformatic analysis are provided in Supplementary Note 1. After quality control, 22,879 cells were analyzed. Uniform manifold approximation and projection (UMAP) identified nine major cell types: epithelial cells, fibroblasts, endothelial cells, T cells, B cells, plasma cells, macrophages, dendritic cells, and mast cells (Fig. 2A-C). T cells constituted the largest fraction of the cellular landscape in both the tumor and lymph nodes (Fig. 2B), suggesting a significant role of T-cell-mediated immunity in the tumor response to afatinib.

We further examined T cells by dividing them into seven subsets: naïve T cells (Naïve_T), double-negative T cells (DN_T), regulatory CD4^+^ T cells (CD4_Treg), exhausted CD4^+^ T cells (CD4_Tex), CD8^+^ effector T cells (CD8_Te), CD8^+^ memory T cells (CD8_Tm), and NK-like T cells (NK_T) (Fig. 2D). Clonal TCR analysis revealed that the hilar lymph nodes had the highest T-cell clonal diversity, whereas the tumors had the greatest clonal expansion (Fig. 2F-G). Highly expanded T-cell clones were predominantly CD8^+^ effector and CD8^+^ memory T cells (Fig. 2E and H).

**Fig. 3:**
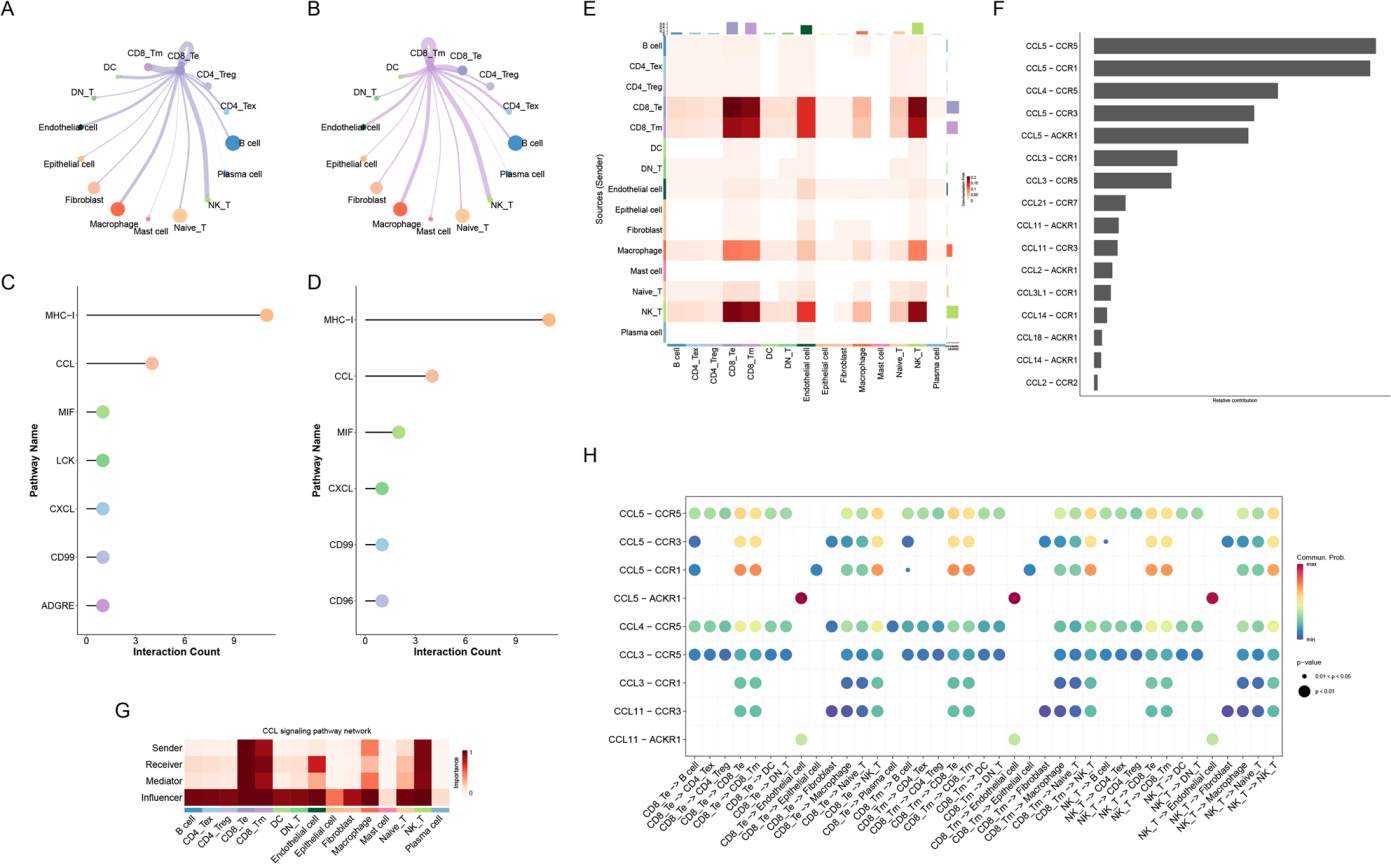
Cell-cell communication analysis. (**A**, **B**) Circle plots of intercellular signaling weights from CD8⁺ effector (**A**) and CD8⁺ memory (**B**) T cells to other cell types. (**C**, **D**) Lollipop charts of the top signaling pathways initiated by CD8⁺ effector (**C**) and CD8⁺ memory (**D**) T cells. (**E**) Heatmap of the CCL ligand-receptor signaling network. (**F**) Bar plot of the contribution of each ligand-receptor pair in the CCL pathway. (**G**) Heatmap indicating each cell group’s signaling role (sender vs. receiver) and their major contributing signals. (**H**) Bubble plot of all significant ligand-receptor interactions involving CD8⁺ effector T cells, CD8⁺ memory T cells, and NK-like T cells. CCL, C-C chemokine ligand; NK-like, natural killer-like

We then used CellChat to infer cell-cell communication among the immune subtypes. CD8^+^ effector and CD8^+^ memory T cells exhibited strong intercellular signaling, predominantly via the C-C motif chemokine ligand (CCL) chemokine pathway (Fig. 3A-D). In particular, CD8^+^ effector T cells, CD8^+^ memory T cells, and NK-like T cells were highly involved in the CCL signaling network, mainly recruiting other T cells through three ligand-receptor pairs: CCL5-C-C chemokine receptor (CCR)5, CCL5-CCR3, and CCL5-CCR1 (Fig. 3E-H). These findings indicate that the tumor microenvironment following afatinib treatment is characterized by the clonal expansion of CD8 + T cells and the presence of cytotoxic T cells recruited through coordinated chemokine signaling.

## Discussion and conclusions

Neoadjuvant-targeted therapies for oncogene-driven NSCLC are an emerging area of investigation [10–12]. Meta-analyses and prospective trials have suggested that neoadjuvant epidermal growth factor receptor (EGFR)-TKI therapy is a feasible treatment modality with favorable surgical outcomes in resectable EGFR-mutant NSCLC. A recent phase II trial of neoadjuvant afatinib in stage III EGFR-mutant NSCLC reported an objective response rate of 70.2% and an R0 resection rate of 87.9% [13]. In our patient, neoadjuvant afatinib induced a marked radiographic response and converted an initially unresectable (cT2aN3M0) tumor into one that was amenable to complete resection.

To the best of our knowledge, only one other report has described the NPTN-NRG1 fusion in lung cancer [8]. Nie et al. reported a patient with metastatic lung adenocarcinoma harboring an NPTN-NRG1 fusion who achieved a progression-free survival of 14 months on fourth-line afatinib. Unlike advanced cases, our patient had stage III disease and was treated in a neoadjuvant setting. Both cases illustrate that NPTN-NRG1-positive tumors can be sensitive to afatinib, thereby extending the relevance of NRG1 fusions beyond typical mucinous histology. Our findings align with previous observations that other NRG1 fusion-driven tumors respond to pan-ErbB inhibitors.

The selection of the neoadjuvant regimen for this Stage IIIB (N3) patient involved a careful evaluation of standard versus biology-driven strategies. While concurrent chemoradiotherapy followed by sequential consolidation immunotherapy represents the standard of care for locally advanced NSCLC, several factors favored the use of afatinib. First, *NRG1* fusions are typically associated with low tumor mutational burden and low PD-L1 expression, characterizing them as ‘immune-cold’ tumors with limited sensitivity to immune checkpoint inhibitors [14]. Consequently, the potential benefit of neoadjuvant chemo-immunotherapy was considered suboptimal. Second, the global eNRGy1 registry has demonstrated that afatinib, a pan-ErbB inhibitor, exerts potent antitumor activity against *NRG1* fusion-positive cancers by blocking the heterodimerization of HER2/HER3 [14]. Finally, the decision was guided by a multidisciplinary team consensus and shared decision-making. Considering the patient’s strong preference for surgery and our institution’s extensive experience in conversion therapy (successfully downstaging initially unresectable cases to resectability), we prioritized this targeted induction strategy. This approach ultimately proved successful, enabling a radical resection.

However, approximately 35% of the tumor cells remained viable in the post-operative surgical specimen, a finding consistent with the trial’s observation that single-agent TKI therapy often leaves residual disease. This finding stands in contrast to the radiographic regression observed preoperatively. Such discrepancy between radiographic response and pathologic response is a well-recognized phenomenon in neoadjuvant TKI therapy. For example, in the EMERGING (CTONG1103) and NEOS trials involving EGFR-mutant NSCLC, neoadjuvant TKI treatment yielded high radiographic response rates but relatively modest Major Pathologic Response rates compared to immunotherapy-based regimens [15, 16]. This may be attributed to the predominantly cytostatic or pro-apoptotic mechanism of TKIs, which differs from the rapid cytotoxic necrosis induced by chemotherapy or immunotherapy. Consequently, the patient underwent complete surgical resection and received adjuvant therapy. To date, our patient remains disease-free on reduced-dose adjuvant afatinib.

In this case, longitudinal ctDNA monitoring served as a critical tool for guiding risk-adapted management. The sustained negative ctDNA status, indicative of deep molecular remission. The sustained absence of ctDNA at two assessment points validated the sufficiency of afatinib monotherapy in the neoadjuvant period, allowing us to forgo treatment escalation and spare the patient from additional toxicity.

Accumulating evidence indicates that the tumor immune microenvironment plays a pivotal role in modulating immune responses and impacting therapeutic efficacy [17–19]. Therefore, in this case study, we employed scRNA-seq to investigate the TME. Our single-cell analysis revealed a robust T-cell response in tumor and lymph node microenvironments. The tumor and lymph nodes exhibited extensive clonal expansion of CD8^+^ effector and memory T cells, whereas the subcarinal lymph nodes (which harbored a metastatic tumor) showed relatively limited expansion, possibly due to tumor-induced immunosuppression. Cell-cell interaction analysis indicated that CD8^+^ effector T cells, CD8^+^ memory T cells, and NK-like T cells engage in mutual recruitment via CCL5-CCR5/CCR3/CCR1 signaling. This suggests that the therapeutic response to afatinib was accompanied by robust T cell infiltration and chemokine-mediated recruitment, in addition to its direct anti-proliferative effects. This finding also aligns with a recent phase II study demonstrating that afatinib treatment leads to T cell activation in the tumor microenvironment of responders [13]. The prominent CCL5-CCR5 interaction observed between T cell subsets suggests an active recruitment mechanism within the tumor microenvironment, which may be mechanistically linked to the therapeutic efficacy of afatinib. Previous study reported that EGFR-TKI treatment induces a tumor cell-intrinsic interferon-like transcriptional response in lung cancer. This TKI-induced program is characterized by the upregulation of chemokines, including CXCL10 and CCL5 [20]. These findings highlight a potential interplay between afatinib and the immune microenvironment that warrants further investigation.

While intriguing, these insights must be interpreted with caution. A primary limitation of this study is its single-case nature and, more critically, the absence of baseline scRNA-seq and TCR-seq data, which precludes a direct longitudinal comparison. Consequently, we cannot definitively distinguish whether the observed immune features—such as T cell clonal expansion—were induced by afatinib or represented pre-existing characteristics of the tumor microenvironment. Therefore, our findings should be viewed as generating a hypothesis regarding the immunomodulatory potential of afatinib, which requires validation in larger, prospective cohorts with paired pre- and post-treatment sampling.

In conclusion, this case suggests that neoadjuvant afatinib is an effective and feasible approach for treating locally advanced NSCLC with NRG1 fusion. Afatinib treatment achieved significant tumor regression with manageable toxicity and did not compromise surgical resection. However, this was a single-patient experience, and the role of afatinib in the neoadjuvant and adjuvant settings for NRG1 fusion-positive NSCLC remains to be defined. Prospective studies are needed to evaluate targeted therapeutic strategies for this rare molecular subset of lung cancer.

## Supplementary Information

Supplementary Material 1.

Supplementary Material 2.

## Data Availability

The datasets used and/or analysed during the current study are available from the corresponding author on reasonable request.

## Abbreviations

NSCLC: Non-small cell lung cancer
NPTN-NRG1: Neuroplastin-neuregulin 1
scRNA-seq: Single-cell RNA sequencing
TCR: T-cell receptor
NK-like: Natural killer-like
CCL5: C-C chemokine ligand 5
CCR5: C-C chemokine receptor 5
ERBB: erb-b2 receptor tyrosine kinase
TKI: Tyrosine kinase inhibitor
CT: Computed tomography
NGS: Next-generation sequencing
ctDNA: Circulating tumor DNA
UMAP: Uniform manifold approximation and projection
