# Supplementary material for: Neoadjuvant afatinib in a patient with locally advanced lung adenocarcinoma harboring an NPTN-NRG1 fusion: a case report
Source: World J Surg Oncol. 2026 Mar 14;24:192. doi: 10.1186/s12957-026-04233-6 (PMC13126915; doi:10.1186/s12957-026-04233-6)
Supplement: Supplementary file 1 — Supplementary Material 1. [file 12957_2026_4233_MOESM1_ESM.docx]

Supplementary Note 1: Materials and Methods for Single-Cell Sequencing

1. Single-cell Library Preparation and Sequencing

Single-cell suspensions were prepared from the fresh tissue samples. The scRNA-seq and scTCR-seq libraries were constructed using the Chromium Single Cell 5’ Reagent Kits (10x Genomics, Pleasanton, CA, USA) according to the manufacturer's user guide. 5’ Gene Expression libraries were prepared to capture the transcriptomic landscape. T cell receptor (TCR) enriched libraries were prepared from the same cDNA using the Chromium Single Cell V(D)J Enrichment Kit (Human T Cell).

The constructed libraries were sequenced on an Illumina platform with a paired-end reading mode.

2. scRNA-seq Data Processing

Raw sequencing data were processed using Cell Ranger (version 7.1.0). The reads were aligned to the human reference genome (GRCh38). Subsequent analysis was performed using the Seurat R package (version 5.0.1). Low-quality cells were filtered based on the following criteria: (1) genes detected < 200 or > 5000; (2) percentage of mitochondrial genes > 25%. The data were normalized using the NormalizeData function, and highly variable features were identified. Principal Component Analysis (PCA) was performed, and cells were clustered using the FindClusters function. Uniform Manifold Approximation and Projection (UMAP) was used for visualization. Cell types were annotated based on the expression of canonical marker genes (e.g., *CD3D*, *CD3E* for T cells; *CD79A* for B cells; *EPCAM* for epithelial cells, etc.).

3. TCR-seq Data Analysis

TCR sequencing data were aligned to the V(D)J reference (GRCh38) using Cell Ranger vdj pipeline. The TCR clonotypes were defined based on the nucleotide sequences of the CDR3 regions of both TCR *α* and *β* chains. TCR clonotype information was added to the Seurat object metadata based on cell barcodes. Clonal expansion was analyzed by calculating the frequency of each unique clonotype within the T cell population.
